# Supplementary material for: Designing of cytotoxic and helper T cell epitope map provides insights into the highly contagious nature of the pandemic novel coronavirus SARS-CoV-2
Source: R Soc Open Sci. 2020 Sep 16;7(9):201141. doi: 10.1098/rsos.201141 (PMC7540743; doi:10.1098/rsos.201141)
Supplement: Supplementary Figure [file rsos201141supp16.doc]

**Venn Diagram of all HLA-II binding epitopes of proteins studied across alleles**


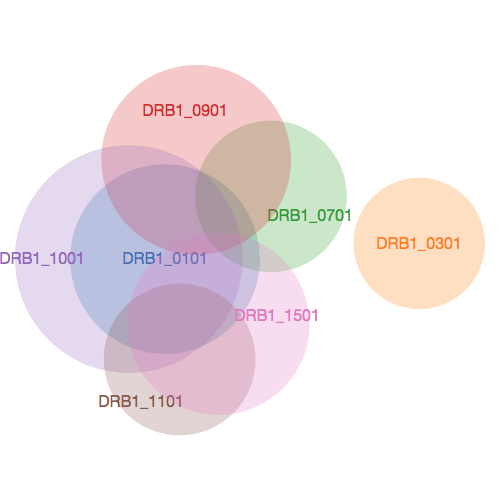
Surface


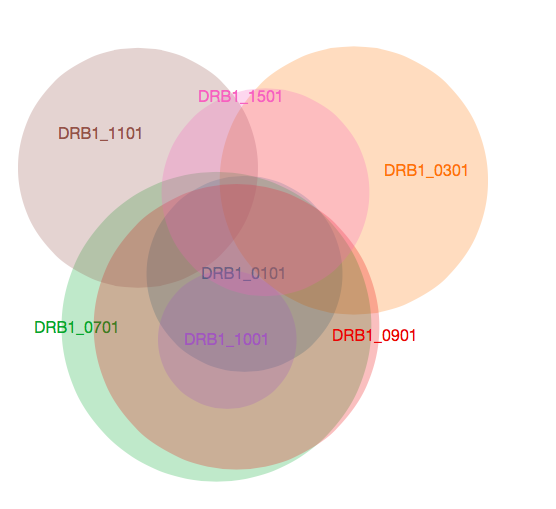
ORF3a


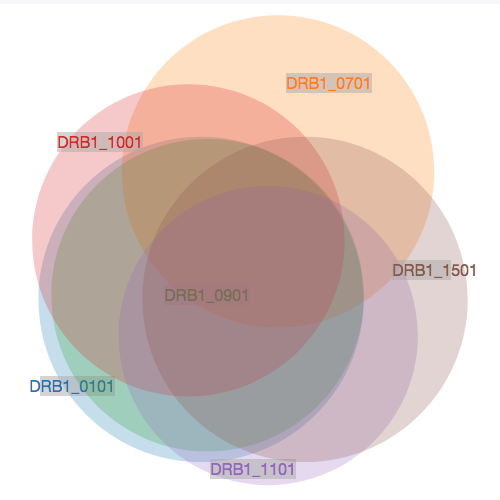


Envelope


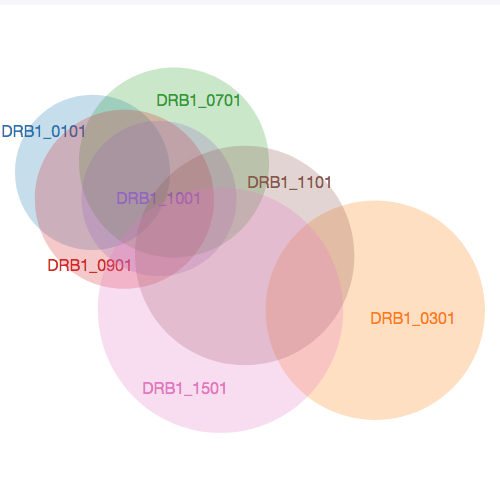
Membrane


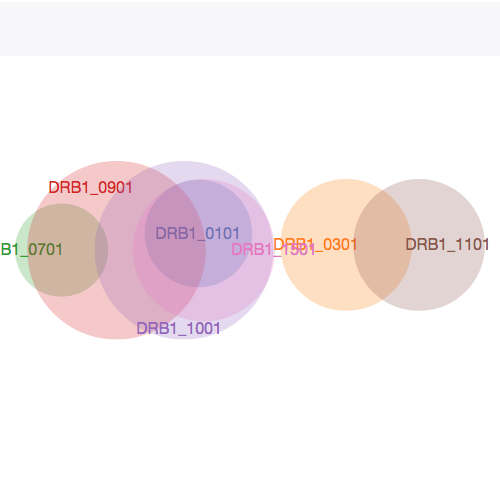
ORF6


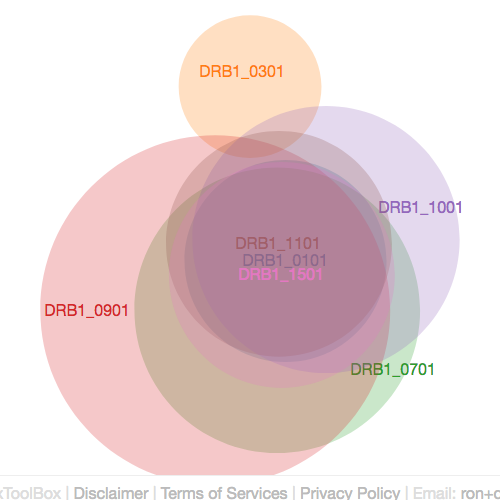
ORF7a


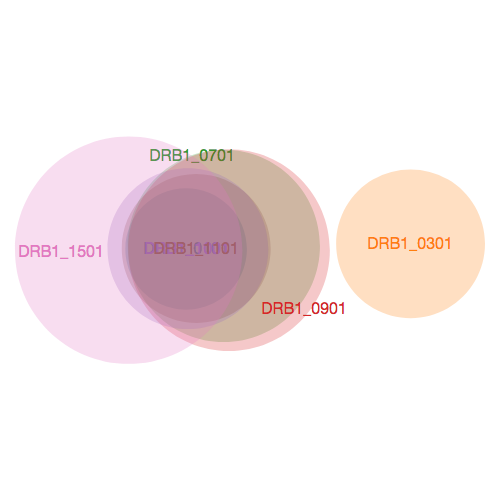
ORF8


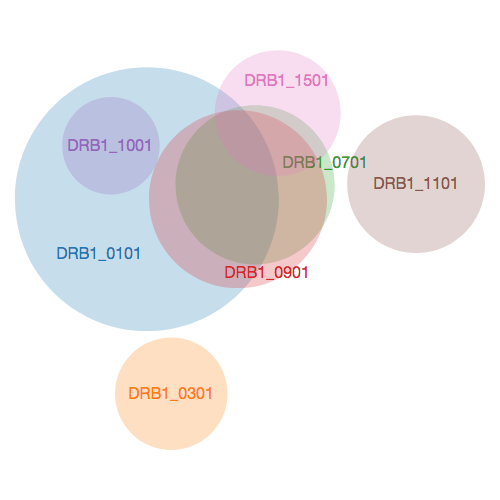


Nucleocapsid


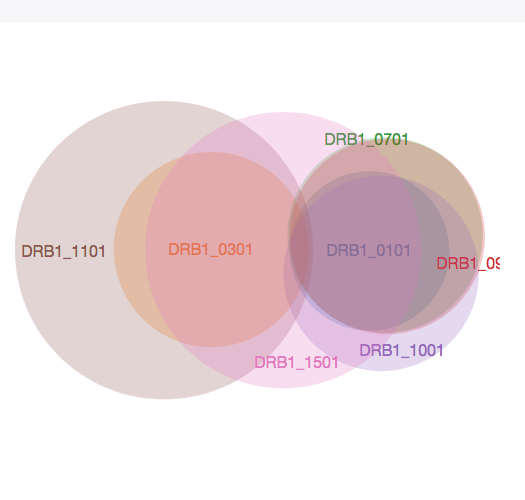


ORF10

ORF1ab proteins:


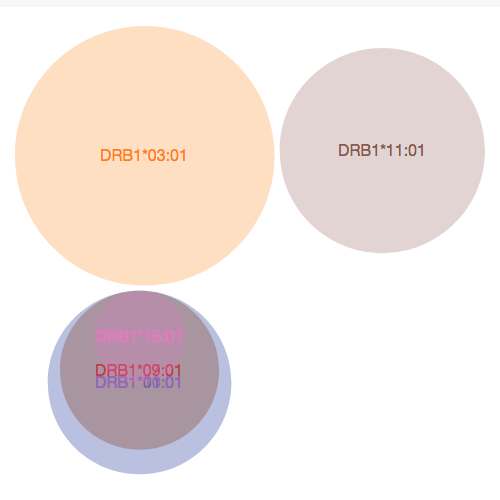


Leader


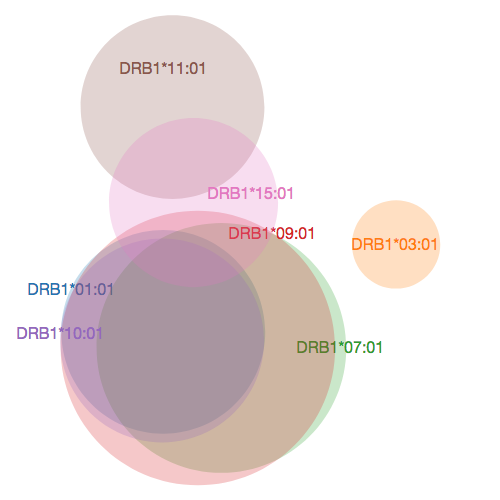


nsp2


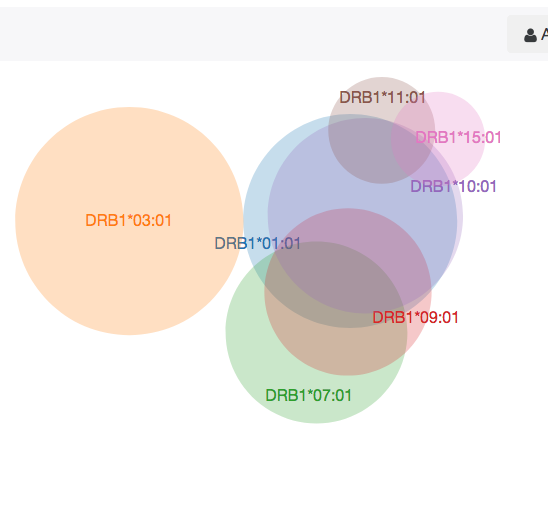
nsp3


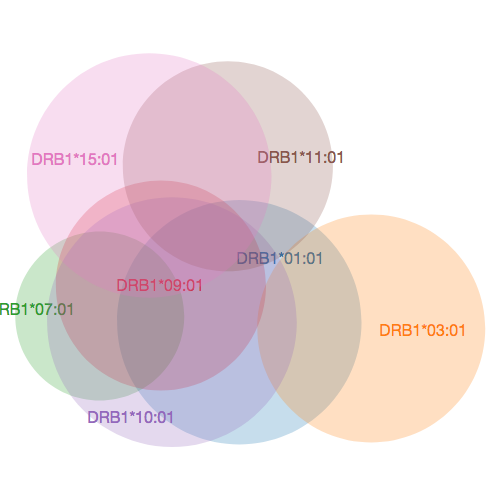
nsp4


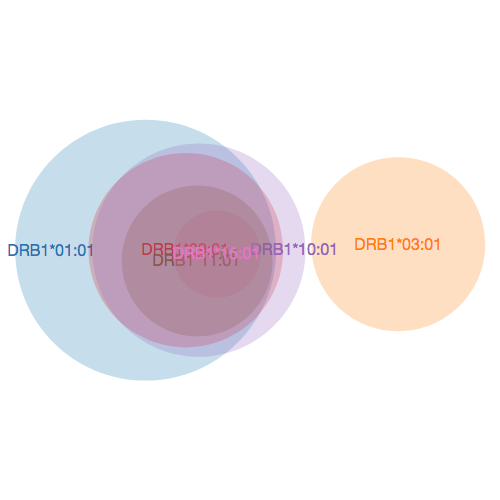
3C-like proteinase


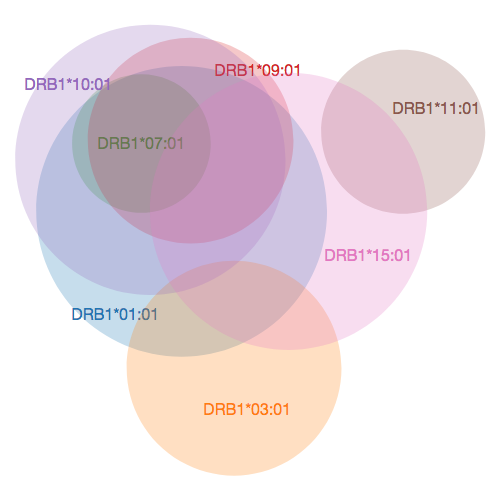
nsp6


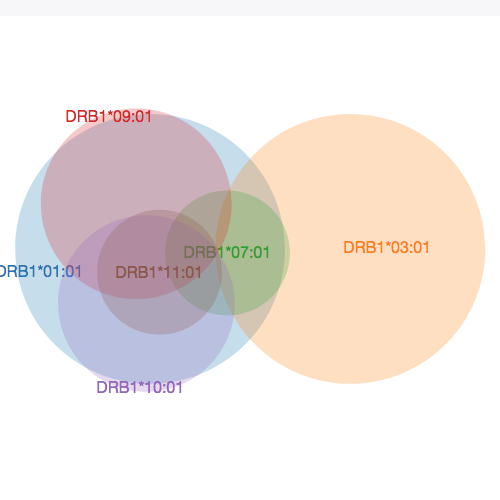
nsp7


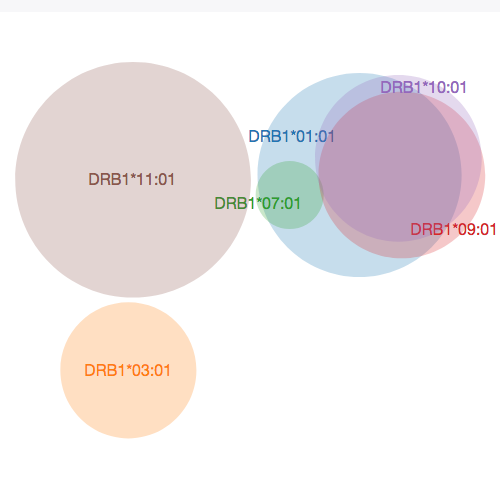
nsp8


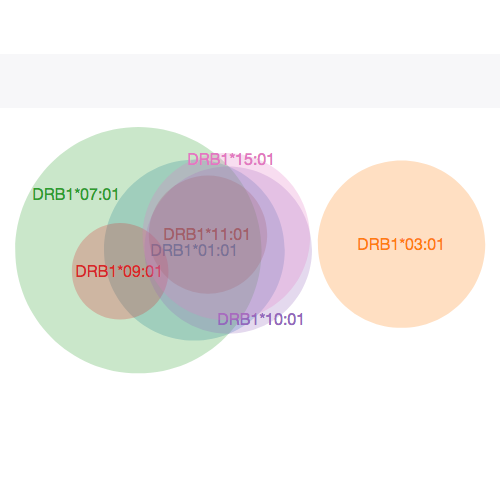
nsp9


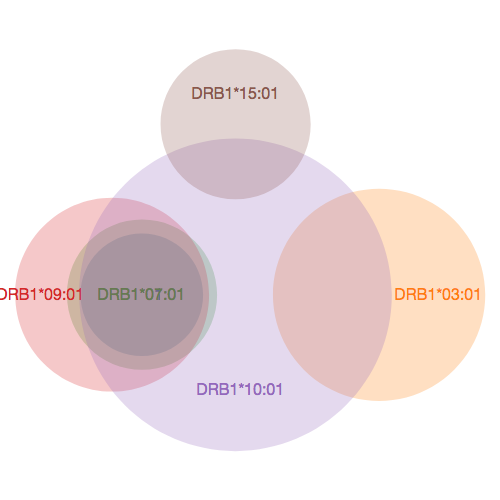
nsp10


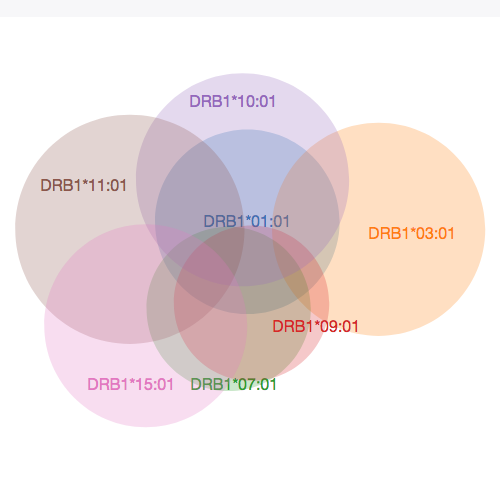
RdRP


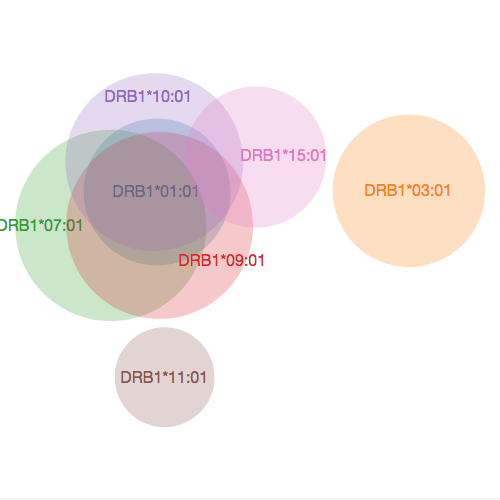
Helicase


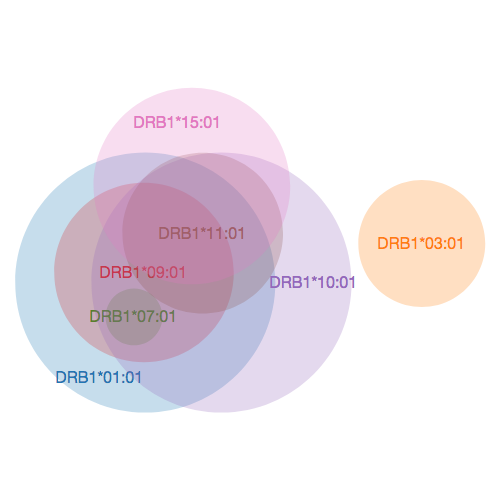
3' to 5'-Exonuclease


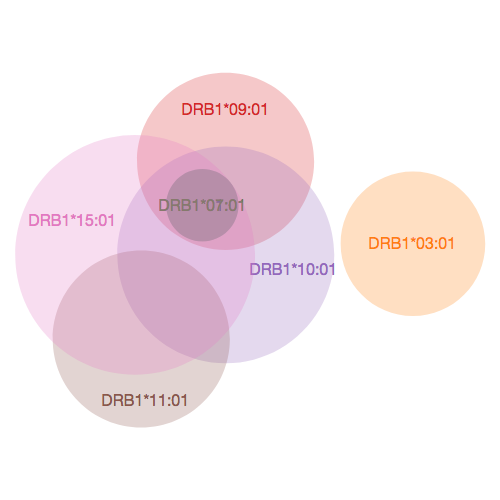
EndoRNAse


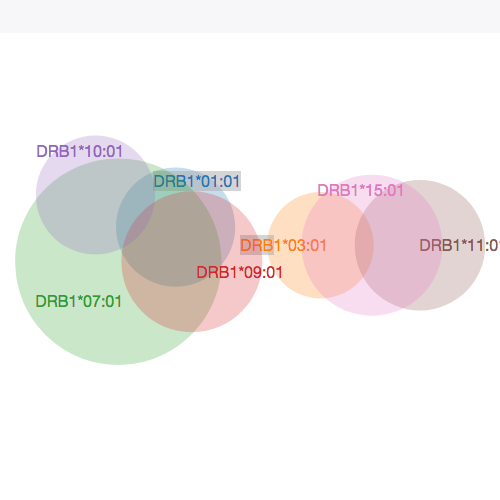


2'-O-ribose methyltransferase
